# Substance use disorder and associated factors among prisoners in a correctional institution in Jimma, Southwest Ethiopia: a cross-sectional study

**DOI:** 10.1186/s12888-018-1901-x

**Published:** 2018-09-27

**Authors:** Yimenu Yitayih, Mubarek Abera, Eliais Tesfaye, Almaz Mamaru, Matiwos Soboka, Kristina Adorjan

**Affiliations:** 1 Department of Psychiatry, College of Health Sciences, Jimma University, Jimma, Ethiopia; 2 Center for International Health, Ludwig-Maximilians-Universität, Munich, Germany; 3 Department of Psychiatry and Psychotherapy, Medical Center of the University of Munich, Munich, Germany; 4 Institute of Psychiatric Phenomics and Genomics (IPPG), Medical Center of the University of Munich, Munich, Germany

**Keywords:** Crime, Prisoners, Substance use disorder, Alcohol use, Khat abuse, Tobacco dependence

## Abstract

**Background:**

Substance use disorder is an important public health problem and one of the major causes of disability worldwide. Substance use and criminal behavior are closely related and there is a significant association between substance misuse and crime, but little is known about substance use disorder among prisoners, in particular in low-income countries. Therefore, we investigated substance use disorder and associated factors in inmates of a correctional institution in Jimma, Southwest Ethiopia.

**Methods:**

We used a cross-sectional study design to collect data from 336 prisoners from June 5 to July 5, 2017. Study participants were selected from the total of 1460 prisoners eligible for the study by a systematic random sampling technique, i.e., one participant was randomly selected from every four consecutive admissions in the registration book. Alcohol use disorder, nicotine dependence, khat abuse, cannabis use disorder, psychopathy, adverse traumatic life events, and social support were assessed. Data were entered into EpiData version 3.1 and analyzed in bivariate and multivariable logistic regression models with the Statistical Package for Social Science version 21. Variables with a *P* value < 0.05 in the final fitting model were declared to be associated with the outcome variable.

**Results:**

The overall prevalence of substance use disorder was 55.9%. The prevalence of khat abuse was 41.9%; alcohol use disorder, 36.2%; nicotine dependence, 19.8%; and cannabis use disorder, 3.6%. Poor social support, living in urban areas, psychopathy, and a family history of substance use were positively associated with substance use disorder.

**Conclusions:**

Substance use disorder is prevalent among prisoners. The increased morbidity and unpleasant psychosocial consequences associated with substance use disorder, together with our finding that 66.3% of prisoners with substance use disorder were interested in obtaining treatment, suggest a need to establish prison-based treatment in this correctional institution in Jimma.

## Background

According to a WHO 2014 report, alcohol consumption, accounted for 5.9% of all deaths worldwide in the year 2012 [1]. Also, smoking caused more than one in ten deaths global, killing more than 6 million people in 2015 [2]. The report also found that the global use of these substances is growing rapidly and contributes significantly to the global burden of disease, assessed as disability-adjusted life years [3, 4]. It estimated that substance use accounted for 14.7% of disability-adjusted life years in 2010 (alcohol: 6.9%) [4].

Substance use and criminal behavior are closely related, and a large proportion of substance users commit crimes while under the influence of a substance [5]. Substance use significantly increases the likelihood of arrest because it increases the need to commit crimes to obtain money to buy a particular substance [5]. Furthermore, 37% of almost 2 million convicted offenders currently in jail were drinking at the time of their arrest [6]. According to a U.S. Department of Justice report, in 2004 about one-third (32%) of inmates in state facilities reported that they had committed a crime while under the influence of drugs [6]. Alcohol is a factor in 40% of all violent crimes in the USA [5], and alcohol and other drugs contribute to 78% of violent crimes, 83% of property crimes, and 77% of public order crimes [7]. Inmates with substance abuse problems are more likely to be re-incarcerated, begin their criminal careers at an early age and have more contact with the criminal justice system [7]. Additionally, prisoners with substance abuse problems are four times more likely to receive income through illegal activities [7].

The risk factors for substance use disorder include a family history of substance use; personality traits, such as high impulsivity or sensation seeking; depression and anxiety; exposure to physical, sexual, or emotional abuse or trauma; and starting substance use at an early age [8]. Rounds-Bryant and Baker [9] stated that ongoing substance abuse in prisoners is a concern because if prisoners are not given adequate treatment in prison and supervision is lacking in the community after they are discharged they have a high potential to become re-addicted and commit a crime after their release. Furthermore, community samples do not necessarily reflect samples in custodial settings, where histories of drug and alcohol use are particularly high compared with the general population [9]. Because a better understanding of this situation could help to highlight the need for different services to meet prisoners’ needs, a systematic review studied the prevalence of problematic substance use and types of substance use disorder diagnosis among people admitted to prison [10].

The review found that 24% of the studied population met the criteria for alcohol use disorder (AUD) and 30% of male and 51% of female prisoners had a drug use disorder [10].

Prison-based drug treatment is diverse and includes a wide range of treatment programs, such as psychosocial-behavioral interventions, therapeutic communities, and victim impact panels, interventions involving legal sanctions, and group and individual psychotherapy for drug-abusing offenders [11]. One study found that 20.5% of prisoners who had completed in-prison residential treatment used drugs or alcohol compared with 36.7% of untreated prisoners within the first six month after release [12].

Despite the contribution of substance use disorder to the global burden of diseases and the high prevalence among incarcerated people, little attention is given to this disorder in the general population and among prisoners in particular [9]. This is particularly true in low-income countries; in Ethiopia, for example, no study has examined the prevalence of substance use disorder in a prison population. Therefore, this study aimed to assess the prevalence of this disorder and associated factors among prisoners in a correctional institution in Jimma, Southwest Ethiopia.

## Methods

### Study setting

The prison is managed by the Oromiya Regional Correcting Units Administrative Office. It was put into service after the expulsion of the occupying Italian forces in 1943 and serves the following regions: Oromiya, Southern nations and nationalities, and Gambella region. The facility was built to accommodate 450 prisoners but currently houses about 1460 (1418 male and 42 female). It is designated as a maximum-security facility. The prison population includes offenders on remand and people convicted to a limited or life-long sentence. The prison compound houses a medical clinic and sick bay, in addition to the usual prison facilities. The study was conducted from June 5 to July 5, 2017.

### Study design

This was a cross-sectional study conducted in the correctional institution in Jimma, Southwest Ethiopia

### Sampling technique

We used a systematic random sampling technique to select participants. The total number of prisoners was used as a sampling frame. About 1460 prisoners were eligible for the study, and the sampling interval was 1460/336 = 4. The first prisoner was randomly selected from the first four prisoners listed in the registration book according to their date of entry into the prison. We then continued to select one prisoner from every subsequent group of 4 until the required sample size was reached (*n* = 336).

### Sample size

The sample size (n) was calculated by the single population proportion formula, *n* = (Zalpha/2)^2^ * P (1-P)/d^2^ [13], assuming a prevalence (P) of 50%, i.e. 0.5 (because a priori prevalence of 0.5 yields a maximally conservative estimate for the required sample size and no similar study has been performed among a prison population in Ethiopia or any other African country), a 95% confidence interval (CI) of 1.96 (Zalpha/2 = 1.96), a 5% margin of error (d, 0.05), and a non-response rate of 10%.

We applied the single population proportion formula to give n = (1.96)^2^ * [0.5 (1–0.5)]/0.05^2^ = 384. Because the population size is < 10,000, we used the finite population correction formula with the calculated sample size of 384 and the total population of 1460:

$$ {\mathrm{n}}_{\mathrm{f}}=\frac{n_i}{1+\frac{n_i}{N}}=384/\left(1+\left[384/1460\right]\right)=305 $$

Thus, assuming a 10% non-response rate the final sample size was 336.

### Study procedures

Data were collected by five Master of Science in Psychiatry students. All data collectors were given two-day training on the study objectives, data collection methods, tools, methods for maintaining confidentiality, acquisition of informed consent, and handling of ethical issues. The five students were supervised by two Master of Science in Public Health students and the principal investigator. A pre-test was conducted on 5% of the sample in the Agaro prison, which is located 45 km from Jimma. On each day of data collection, the completed questionnaires were checked for completeness. The collected data were entered into a computer and processed in a timely fashion.

We assessed the presence of four substance use disorders: AUD, khat abuse, nicotine dependence, and cannabis use disorder. To identify AUD, we used the Alcohol Use Disorders Identification Test (AUDIT) [14]. An AUDIT score ≥ 8 is considered to indicate an AUD. The sensitivity and specificity of AUDIT for AUD are 0.90 and 0.80, respectively [14], and the reliability in this study was 0.87 (Cronbach’s α). We assessed nicotine dependence with the Fagerstrom Test for Nicotine Dependence (FTND), in which a score ≥ 1 indicates nicotine dependence [15]. The reliability of the FTND in this study was 0.80 (Cronbach’s α). To evaluate khat abuse, we used the Drug Abuse Screening Test (DAST); a score ≥ 3 indicates abuse [16]. The reliability of the DAST in this study was 0.88 (Cronbach’s α). Finally, we used the Cannabis Use Problems Identification Test (CUPIT) to assess cannabis use disorder; a score ≥ 12 indicates a cannabis use disorder [17]. The reliability of CUPIT in this study was 0.87 (Cronbach’s α). We chose those tools to assess substance use disorder because they were previously used in similar populations [18].

We used a questionnaire to assess the following potential explanatory variables for substance use disorder: socioeconomic factors (age, sex, marital status, ethnicity, religion, educational status, occupation, income); environmental factors (family history of substance use, social support, immigration history); behavioral and mental health factors (previous known mental illness, perception that substance use does not impair health, start of substance use at an early age, chronic physical illness, suicidal ideation and attempts); and criminal factors (previous arrest, previous substance-related offences, type of crime, committed a crime under the influence of a substance). We chose to explore these variables because an earlier study found that they are relevant for substance use disorder [7, 19]. We also assessed social support with the Oslo 3-item Social Support Scale [20], psychopathy with the Psychopathy Checklist: Screening Version (PCL: SV; cut-off score ≥ 13 [21]; sensitivity, 0.94; specificity, 0.85; reliability in this study: Cronbach’s α = 0.86), and adverse traumatic life events with the Life Events Checklist (positive if at least one traumatic event recorded).

Lastly, we used a 4-item questionnaire to gather data on other aspects of substance use: 1. which reason(s) explain(s) why you use the substance? (possible responses: [A] To relax; [B] To relieve stress; [C] To be accepted by peers (peer pressure); [D] To feel normal; [E] For confidence to commit an offence; [F] Others [specify]); 2. Have you been treated for a substance use disorder? ([A]Yes; [B] No); 3. Are you interested in receiving treatment for substance use disorder? ([A] Yes; [B] No); 4. Are you interested in continuing to take the substance after you are released from prison? ([A] Yes; [B] No).

### Data processing and analysis

EpiData Version 3.1 was used to enter the data; the mean was used in case of missing data. Then, the data were exported to Statistical Package for Social Science version 21.0 for further analysis. Descriptive statistics, such as the frequency and median, were computed, and bivariate and multivariable analyses were used to identify factors associated with the outcome variable. Factors associated with the outcome variables that had a *P* value < 0.25 in the bivariate analysis were included in the multivariable analysis. Statistical significance was set at *P* < 0.05. An odds ratio with a 95% CI was computed to assess the level of association and statistical significance.

### Ethical considerations

The study protocol was approved by the Research Ethics and Approval Committee of the Jimma University Institute of Health, and the study was performed according to the Declaration of Helsinki. Verbal informed consent was obtained from prisoners. Participants were told that selection for participation in the study was random and that they had the right not to respond to questions they were not comfortable with and to ask questions. After data entry was complete, the questionnaires were kept securely locked away.

## Results

### Socio-demographic characteristics

A total of 329 prisoners participated in the study. The response rate was 97.9%: of the 336 prisoners approached to participate in the study, *n* = 7 (2.1%) declined to participate because they were unwilling to be interviewed about their substance use histories. The median age of the participants was 26 years (inter-quartile range [IQR] 14). The majorities of participants had been residing in urban areas before imprisonment and were unmarried. The most common ethnicity was Oromo, and the most common religion, Muslim. Detailed information on socio-demographic characteristics is given in Table 1.

**Table 1:** Socio-demographic characteristics of prisoners in the correctional institution in Jimma, Southwest Ethiopia, June–July 2017 (*n* = 329) and bivariate analysis of socio-demographic characteristics

Variables	Category	Frequency	Percentage (%)	Substance use disorder		COR (95% CI)	*P* value
				No (%)	Yes (%)		
Sex	Male	307	93.3	130 (42.3)	177 (57.7)	2.92 (1.16–7.36)	0.023^a^
	Female	22	6.7	15 (68.2)	7 (31.8)	Reference value	
Age categories	< 30	219	66.6	98 (44.7)	121 (55.3)	1.52 (0.49–4.67)	0.465
	≥ 30	110	33.4	47 (42.7)	63 (57.3)	Reference value	
Place of residence	Rural	106	32.2	60 (56.6)	46 (43.4)	Reference value	
	Urban	223	67.8	85 (38.1)	138 (61.9)	2.12 (1.32–3.39)	0.002^a^
Ethnicity^a^	Oromo	210	63.8	–	–	–	–
	Amhara	51	15.5	–	–	–	–
	Tigray	16	4.9	–	–	–	–
	Gurage	17	5.2	–	–	–	–
	Dawuro	17	5.2	–	–	–	–
	Yem	16	4.9	–	–	–	–
	Other^b^	2	0.6	–	–	–	–
Educational status	No formal education	27	8.2	11 (40.7)	16 (59.3)	Reference value	
	Primary education	178	54.1	83 (46.6)	95 (53.4)	0.79 (0.35–1.79)	0.568
	Secondary education	94	28.6	41 (43.6)	53 (56.4)	0.89 (0.37–2.12)	0.790
	Tertiary education	30	9.1	10 (33.3)	20 (66.7)	1.38 (0.47–4.05)	0.563
Religion	Muslim	181	55.0	79 (43.6)	102 (56.4)	2.91 (0.86–9.78)	0.085^a^
	Orthodox	97	29.5	40 (41.2)	57 (58.8)	3.21 (0.92–1.14)	0.067^a^
	Protestant	38	11.6	17 (44.7)	21 (55.3)	2.78 (0.73–0.62)	0.135^a^
	Catholic	13	4.0	9 (69.2)	4 (30.8)	Reference value	
Marital status	Married	124	37.7	58 (46.8)	66 (53.2)	Reference value	
	Unmarried	205	62.3	87 (42.4)	118 (57.6)	1.19 (0.76–1.87)	0.443
Occupation	Employed	134	40.7	62 (46.3)	72 (53.7)	Reference value	
	Unemployed	36	10.9	14 (38.9)	22 (61.1)	1.35 (0.64–2.87)	0.430
	Farmer	99	30.1	40 (40.4)	59 (59.6)	1.27 (0.75–2.15)	0.373
	Student	43	13.1	22 (51.2)	21 (48.8)	0.82 (0.41–1.64)	0.576
	Other^c^	17	5.2	7 (41.2)	10 (58.8)	1.23 (0.44–3.43)	0.692
Average monthly income (birr)	< 1200	192	58.4	81 (42.2)	111 (57.8)	Reference value	
	≥1200	137	41.6	64 (46.7)	73 (53.3)	0.83 (0.54–1.29)	0.415

Reference value: In the analysis, this variable indicated lower risk for developing substance use; coded as zero in SPSS logistic regression

^a^Bivariate analysis was not performed for ethnicities

^b^Other ethnicities: kafa, walayita, and silte

^c^Other occupations: retired or homemaker

### Prevalence of substance use disorder

A total of *n* = 227 (69.0%) of the participants had a history of substance use. More than half of all participants (*n* = 184/329, 55.9%) reported a substance use disorder within the 12 months before imprisonment, and half (*n* = 165/329, 50.2%) had used a substance within the 30 days before imprisonment.

Khat was the most commonly used substance in the 12 months before imprisonment, (*n* = 138/329, 41.9%). Among the participants with khat abuse, *n* = 81(58.7%) had harmful use, and *n* = 57 (41.3%), khat dependence. The prevalence of AUD within the 12 months before imprisonment was 36.2% (*n* = 119/329). Among the participants with an AUD, *n* = 57 (47.9%) had hazardous drinking; *n* = 20 (16.8%), harmful drinking; and *n* = 42 (35.2%), alcohol dependence. The next most commonly used substance was nicotine: *n* = 65/329 (19.8%) of participants had a history of nicotine dependence within the 12 months before imprisonment (low dependence: *n* = 28, 43.1%; low to moderate dependence: *n* = 10, 15.4%; moderate dependence: *n* = 22 (33.8%); and high dependence: *n* = 5 (7.7%). The prevalence of cannabis use disorder within the 12 months before imprisonment was 3.6% (*n* = 13/329).

A total of *n* = 105 participants (31.9%) had a history of two or more substance use disorders. Among the prisoners with an AUD, *n* = 87 (73.1%) had a history of khat abuse; *n* = 41 (34.5%), a history of nicotine dependence; and *n* = 13 (10.9%), a history of cannabis use disorder. Among the prisoners with khat abuse, *n* = 87 (63%) had a history of AUD; *n* = 43 (31.2%), a history of nicotine dependence; and *n* = 13 (9.4%), a history of cannabis use disorder. Among the prisoners with nicotine dependence, *n* = 43 (66.2%) had a history of khat abuse; *n* = 41 (63.1), a history of AUD; and *n* = 12 (18.5%), a history of cannabis use disorder.

The median age at the first use of a substance was 16 years (IQR 5), and 44% of the participants started using the substance before the age of 15 years. The median duration of substance use was 6 years (IQR 7).

The main reasons for starting substance use reported by participants were peer pressure (*n* = 77, 41.8%), recreational reasons (*n* = 63, 34.2%), and stress relief (*n* = 54, 29.3%). Almost all of the prisoners with a substance use disorder had not received treatment prior to imprisonment (*n* = 178, 96.7%), but *n* = 122 (66.3%) of them were interested in receiving treatment. A third (*n* = 62, 33.7%), however, wanted to continue using the substance after they were released from prison.

Among the prisoners with a substance use disorder, the most common reasons for imprisonment were assault (*n* = 65, 35.3%) and theft (*n* = 47, 25.5%). The most common reasons for imprisonment among participants with an AUD, nicotine dependence, or cannabis use disorder were assault (*n* = 42, 35.3%; *n* = 24, 36.9%; and *n* = 5, 38.5%, respectively) and murder (*n* = 28, 23.5%; *n* = 12, 26.2%; and *n* = 4, 30.8%, respectively), and among prisoners with khat abuse, assault (*n* = 48, 34.8%) and theft (*n* = 38, 27.5%).

### Prison history

The median age of participants at the first imprisonment was 24 years (IQR 13). Nearly all of the participants were convicted prisoners. At the time of the study, the median duration of incarceration was 48 months (IQR 90) and the median duration of time spent in prison was 9 months (IQR 20).

Assault and murder were the most common causes of imprisonment. Most of the participants had no previous history of imprisonment, and most of the crimes were not committed under the influence of a substance. Among the crimes that were committed under the influence of a substance (*n* = 42/329), *n* = 24 (57.1%) were committed under the influence of alcohol; *n* = 14 (33.3%), under the influence of khat; and *n* = 4 (9.6%), under the influence of cannabis. Table 2 shows the study participants’ forensic history. See (Table 2)

**Table 2:** Forensic history of prisoners (*n* = 329) in the correctional institution in Jimma, Southwest Ethiopia, in June–July 2017

Variables		Frequency	Percentage (%)	Substance use disorder	
				No, *n* (%)	Yes, *n* (%)
Reason for admission to prison	On remand	25	7.6	15 (60.0)	10 (40.0)
	Convicted	304	92.4	130 (42.8)	174 (57.2)
Type of offence committed	Assault	103	31.3	38 (36.9)	65 (63.1)
	Murder	83	25.2	41 (49.4)	42 (50.6)
	Theft	81	24.6	34 (42.0)	47 (58.0)
	Rape	32	9.7	16 (50.0)	16 (50.0)
	Robbery	12	3.6	5 (41.7)	7 (58.3)
	Other^a^	18	5.4	11 (61.1)	7 (38.9)
Previous imprisonment	No	301	91.5	140 (46.5)	161 (53.5)
	Yes	28	8.5	5 (17.9)	23 (82.1)
Committed crime under the influence of substance	No	287	87.2	140 (48.4)	147 (51.6)
	Yes	42	12.8	5 (11.9)	37 (88.1)

^a^Other types of offence = political offence and offence related to forest destruction

### Factors associated with substance use disorder

Various behavioral, mental health, environmental, and criminal factors were found to be associated with substance use disorder (see Table 3).

**Table 3:** Bivariate analysis of behavioral, mental health, environmental, and criminal factors among prisoners in the correctional institution in Jimma, Southwest Ethiopia, June–July 2017 (*n* = 329)

Variable		Substance use disorder		COR (95% CI)	*P* value
		No (%)	Yes (%)		
Psychopathy	No	139 (48.3)	149 (51.7)	Reference value	
	Yes	6 (14.6)	35 (85.4)	5.44 (2.22–13.34)	0.001*
Adverse traumatic life event	No exposure to traumatic life event	62 (51.7)	58 (48.3)	Reference value	
	One traumatic life event	33 (52.4)	30 (47.6)	0.97 (0.53–1.79)	0.927
	Multiple traumatic life events	50 (34.2)	96 (65.8)	2.05 (1.25–3.37)	0.004*
Mental illness	No	132 (44.3)	166 (55.7)	Reference value	
	Yes	13 (41.9)	18 (58.1)	1.10 (0.52–2.33)	0.801
Social support	Poor	53 (28.8)	131 (71.2)	4.35 (2.42–7.81)	0.001*
	Moderate	48 (63.2)	28 (36.8)	1.03 (0.52–2.02)	0.939
	Strong	44 (63.8)	25 (36.2)	Reference value	
Immigration	No	123 (45.6)	147 (54.4)	Reference value	
	Yes	22 (37.3)	37 (62.7)	1.40 (0.79–2.51)	0.248*
Previous imprisonment	No	140 (46.5)	161 (53.5)	Reference value	
	Yes	5 (17.9)	23 (82.1)	4.00 (1.48–10.80)	0.006*
Family history of substance use	No	110 (57.0)	83 (43.0)	Reference value	
	Yes	35 (25.7)	101 (74.3)	3.82 (2.37–6.17)	0.001
Suicidal ideation and suicide attempt	No	114 (46.9)	129 (53.1)	Reference value	
	Yes	31 (36.0)	55 (64.0)	1.57 (0.94–2.60)	0.082*
Chronic physical illness	No	120 (43.5)	156 (56.5)	Reference value	
	Yes	25 (47.2)	28 (52.8)	0.86 (0.48–1.55)	0.62
Perceived that substance use did not affect health	No	66 (46.5)	76 (53.5)	Reference value	
	Yes	79 (42.2)	108 (57.8)	1.19 (0.77–1.84)	0.444

Reference value: In the analysis, this variable indicated lower risk for developing substance use; coded as zero in SPSS logistic regression

*Identified as factors for multivariable logistic regression analysis (*P* < 0.25)

Multivariable logistic regression showed that four variables were significantly associated with substance use disorder: poor social support (adjusted odds ratio [AOR]: 4.41; 95% CI, 2.22–8.77), living in an urban setting (AOR: 2.42; 95% CI, 1.33–4.40), psychopathy (AOR: 4.68; 95% CI, 1.71–12.78), and a family history of substance use (AOR: 4.39; 95% CI, 2.49–7.79). Prisoners with poor social support were more than four times more likely to develop a substance use disorder than prisoners with good social support (AOR:4.41; 95% CI, 2.22–8.77). Also, prisoners living in an urban setting were more than two times more likely to have a substance use disorder than prisoners living in a rural setting (AOR:2.42; 95% CI, 1.33–4.40). Prisoners with psychopathy were nearly five times more likely to develop a substance use disorder than those without psychopathy (AOR: 4.68; 95% CI, 1.71–12.78). Prisoners with a family history of substance use were more than four times more likely to have a substance use disorder than prisoners with no family history of substance use (AOR: 4.39; 95% CI, 2.49–7.79) (see Table 4).

**Table 4:** Multivariable logistic regression analysis for independent predictors of a substance use disorder among prisoners (*n* = 329) in a correctional institution in Jimma, Southwest Ethiopia, in June–July 2017

Variable		Substance use disorder		AOR (95% CI)
		No, *n* (%)	Yes, *n* (%)	
Social support	Poor support	53 (28.8)	131 (71.2)	4.41 (2.22–8.77)
	Moderate support	48 (63.2)	28 (36.8)	1.07 (0.49–2.34)
	Strong support	44 (63.8)	25 (36.2)	Reference value
Family history of substance use	No	110 (57.0)	83 (43.0)	Reference value
	Yes	35 (25.7)	101 (74.3)	4.39 (2.49–7.79)
Psychopathy	No	139 (48.3)	149 (51.7)	Reference value
	Yes	6 (14.6)	35 (85.4)	4.68 (1.71–12.78)
Place of residence	Rural	60 (56.6)	46 (43.4)	Reference value
	Urban	85 (38.1)	138 (61.9)	2.42 (1.33–4.40)

## Discussion

This cross-sectional study assessed the prevalence of substance use disorder among prisoners in a correctional facility in Jimma, Southwest Ethiopia, in the 12 months before imprisonment. The prevalence of substance use disorder was 55.9%, which is similar to a comparable study performed in Australia (66%) [22].

The prevalence of AUD among prisoners in the current study was in line with a similar study performed Sudan (32.2%, *N* = 1569) [23] but lower than the prevalence found in a Finnish study (68%, *N* = 610) [24]. The difference might be due to differences in sample size and the tool used to assess AUD (the Finnish study used a clinical psychiatric interview in a larger sample). A study performed in a prison in Lyon, France, found a lower prevalence of AUD (13.7%, *N* = 535) [25], which may be because it was performed in women only and the participants may have had other differences in socio-demographic characteristics.

The prevalence of khat abuse found in the current study was much higher than that in a study in Uganda (17%) [26]. However, the Ugandan study used a self-report questionnaire, so khat use may have been underreported.

The prevalence of nicotine dependence among the prisoners in the current study in the 12 months before imprisonment was lower than the findings of studies in Kenya (32.7%) [27] and Lyon (37.5%) [25]. The differences might be due to the different tools used to measure nicotine dependence and differences between the study populations (the Kenyan study used a self-report questionnaire, and the study in Lyon was in female participants only).

The presence of psychopathy was significantly associated with substance use disorder. This finding is in line with similar studies performed in England and Wales [28] and Turkey [29], both of which found that psychopathy was associated with substance use disorder. The reason for this association might be that people with psychopathy have an irresponsible lifestyle, are impulsive, curious to try new things and unconventional and show sensation-seeking behavior, which in turn makes them more likely to use a substance as a form of self-medication [28].

In the current study, prisoners who lived in an urban setting had a higher likelihood of developing a substance use disorder than those who lived in a rural setting. This finding is in line with a similar study performed in Kenya [27]. This difference might be explained by various characteristics of urban environments, such as population density, built-up environments, and greater access to substances [30].

Both in our study and in a study performed in incarcerated delinquents in Nigeria [31], a family history of substance use was associated with the prisoners’ substance use disorder. Prisoners with a family history of substance use had a more than four times higher likelihood of developing a substance use disorder than those with no such family history. One reason for this association might be that parents with a history of substance use may not be deeply involved in bringing up their family and may be less attached to their family, so that their children have difficulties in learning social behavior patterns and experience more adverse life events [32]. Furthermore, families may have a genetic susceptibility to substance use, and it may also be a learned behavior. The implication of this finding is that substance use awareness campaigns should target not only prisoners but also their parents and relatives.

Prisoners with poor social support were more likely to develop a substance use disorder than those with good social support. A study performed in Hungary also found that poor social support, in the form of poor support from the father, was a factor for substance use [33]. Social support has been found to help people cope with stress and to reduce the risk for anxiety, depression, and distress, all of which are risk factors for substance use [34]. Thus, people with poor social support might be that at greater risk of using substances. Furthermore, people may be more likely to use substances if they have no one to live for other than themselves. This may also apply to people who do not receive any feedback and criticism from others because such feedback and criticism could help them form socially acceptable behavior.

Our finding that just over half of all prisoners had a substance use disorder within 12 months prior to their imprisonment is alarming in view of the fact that substance use disorder treatment is not accessible in the prison. If current prevalence rates are generalizable to all prisons in Ethiopia, our results suggest that about two thirds of prisoners with substance use disorder are interested in receiving treatment; however, almost no prisoners with substance use disorder have access to such treatment services. With regard to the treatment of substance use disorder in prison, therapeutic community intervention (TCI) and individual and group therapy are methods that can decrease the rates of re-incarceration, drug misuse relapse, and re-arrest [35]. Research has shown that an unfortunate consequence of this shortage of treatment services is that offenders quickly return to drug use and crime after their release from prison [36, 37].

In summary, this study has substantial clinical implications for health services in correctional institutions because it shows the need for management plans for acute substance withdrawal and for recovery and rehabilitation support for prisoners with substance use disorder.

### Limitations

This study may have a social desirability bias, i.e. prisoners may have underestimated, underreported or denied their substance use. Also, data on the previous 12 months were collected by interview, which has a risk of recall bias. DAST was not validated in our population, even though it has been shown to be useful in screening for khat abuse across cultures. Another limitation is that prisoners’ reports of past or present physical illness and mental illness were not clinically confirmed.

## Conclusions

This study found a high prevalence of substance use disorder among prisoners in a correctional institution in Jimma, Southwest Ethiopia, in the 12 months before imprisonment. The most commonly used substance was khat, followed by alcohol, nicotine, and cannabis. Living in an urban setting and having psychopathy, a family history of substance use, and poor social support were positively associated with substance use disorder. Generally, despite the increased morbidity of substance users and unpleasant psychosocial consequences of this habit, most prisoners reported not receiving treatment prior to imprisonment. The large number of prisoners with substance use disorder, the unavailability of treatment in prisons, and the substantial gap in services relative to the need indicate that prisoners are more likely to return to risky substance use after release from prison. Therefore, increasing access to substance use disorder treatment, such as TCI, in prison could have substantial long-term economic and social benefits, e.g. reduced recidivism, easier transition to the community after release, and less drug abuse [38].

Further research is needed into substance abuse in other correctional institutions in Ethiopia and the efficacy of prison-based treatment for substance use disorder.

## Abbreviations

AOR: Adjusted odds ratio
AUD: Alcohol use disorder
AUDIT: Alcohol Use Disorders Identification Test
CUPIT: Cannabis Use Problems Identification Test
DAST: Drug Abuse Screening Test
FTND: Fagerstrom Test for Nicotine Dependence
IQR: Interquartile range
